# Full-Length Transcriptome Reveals Alternative Splicing Regulation and Key Genes Underlying Differential Ginsenoside Accumulation Between Wild-Simulated and Cultivated Ginseng

**DOI:** 10.3390/biology15161367

**Published:** 2026-08-11

**Authors:** Xinyang Liu, Xinyi Zhao, Han Wang, Xiaoshuang Wei, Mengmei Hu, Yang Liu, Zhennan Wang, Dan Zhao, Zhenhui Wang

**Affiliations:** 1 Faculty of Agronomy, Jilin Agricultural University, Changchun 130118, China; liuxinyang1219@163.com (X.L.); 18844065830@163.com (X.Z.); wanghan19960307@163.com (H.W.); weixiaoshuang@jlau.edu.cn (X.W.); mengmeihu1997@163.com (M.H.); liuyuxi5389@163.com (Y.L.); zhongdide1982@163.com (Z.W.); 2 Ginseng and Antler Products Testing Center of the Ministry of Agricultural and Rural Affairs of the People’s Republic of China, Jilin Agricultural University, Changchun 130118, China

**Keywords:** wild-simulated ginseng, ginsenoside, full-length transcriptome, alternative splicing, WGCNA

## Abstract

Ginsenoside accumulation is tissue- and germplasm-specific, yet its transcriptional regulation remains unclear. We combined HPLC, PacBio full-length transcriptome, and Illumina RNA-seq to compare 4-year cultivated ginseng with 26-year wild-simulated ginseng. After clustering and error correction, we obtained 265,373 (CG) and 210,343 (WSG) non-redundant full-length transcript isoforms. Total saponins ranked leaves > roots > stems. In leaves, PPT type (Re and Rg1) predominated and was significantly higher in CG than WSG; in roots, PPD type dominated, and all monomer saponins in WSG were significantly higher. Transcriptome analysis revealed widespread alternative splicing (mainly retained introns), with key genes like *CYP716A47* showing differential expression and splicing. WGCNA identified a yellow module positively correlated with PPD-type ginsenosides, enriched in terpenoid and CYP/UGT pathways and containing *CYP716A47* and multiple UGT candidates. qRT-PCR validated 12 selected genes (6 known, 6 novel). Our findings reveal multilevel transcriptional and post-transcriptional regulation associated with the distinct production backgrounds of cultivated and wild-simulated ginseng and provide candidate targets for future functional studies and metabolic engineering.

## Simple Summary

*Panax ginseng* produces ginsenosides as its main active compounds, but variation in ginsenoside accumulation associated with cultivation background, plant age, and growth environment remains incompletely understood. We compared 4-year cultivated ginseng (CG) and 26-year wild-simulated ginseng (WSG) in roots, stems, and leaves using PacBio and Illumina sequencing, metabolite profiling (HPLC), and WGCNA. WSG had higher ginsenoside levels and showed distinct transcript isoform profiles compared with CG. Enhanced accumulation in WSG was linked to upregulated downstream genes and altered splicing patterns. These findings offer genomic resources and candidate genes for breeding.

## 1. Introduction

*Panax ginseng* C. A. Meyer has been a mainstay of East Asian traditional medicine for over two millennia [1,2]. Its wide-ranging therapeutic effects—including anticancer, neuroprotective, immunomodulatory, and anti-diabetic activities—are mainly attributed to ginsenosides, a structurally diverse class of triterpenoid saponins [3,4]. Over 150 ginsenosides have been identified from *Panax* species, with protopanaxadiol (PPD)- and protopanaxatriol (PPT)-type ginsenosides being the most abundant and bioactive [5,6]. Elucidating the molecular basis of their biosynthesis and tissue-specific distribution remains a central goal [5], and high-performance liquid chromatography–mass spectrometry (HPLC-MS) profiling serves as a key tool for characterizing their spatial distribution patterns [7]. Ginseng cultivation falls into two primary categories: cultivated ginseng and wild-simulated ginseng [8]. Wild-simulated ginseng displays superior pharmacological activities, including stronger anti-aging effects, is enriched in rare ginsenosides (Rk1, F1, Rg5, Rh1, and CK) and distinct phytosterols such as stigmasterol [9,10,11], and shows higher levels of volatile compounds including hexanal and heptanal [12].

Ginsenosides are triterpenoids derived from the isoprenoid pathway. The mevalonate (MVA) pathway supplies IPP and DMAPP, which are subsequently converted into 2,3-oxidosqualene, the universal precursor of triterpenoid saponins [13,14]. Cyclization of this intermediate by oxidosqualene cyclases (OSCs) represents a critical rate-limiting step: dammarenediol-II synthase (DDS) generates tetracyclic dammarenediol-II, whereas β-amyrin synthase (β-AS) produces pentacyclic β-amyrin for oleanane-type saponins [13]. Subsequent hydroxylation and carboxylation by cytochrome P450s (CYPs) and glycosylation by UDP-glycosyltransferases (UGTs) determine the final structures and bioactivities of ginsenosides [13,14,15,16,17]. Although a limited number of CYP450 and UGT family members have been functionally characterized, most remain unannotated, and their evolutionary expansion warrants systematic investigation [15,16,18,19,20]. Co-expression of *PgDDS* and *CYP716A47* in yeast yields protopanaxadiol without the addition of exogenous substrates [16], underscoring the pivotal roles of these enzymes. Their transcriptional regulation may directly influence ginsenoside accumulation.

Transcriptomic and metabolomic studies have extensively examined how growth duration affects ginsenoside accumulation in cultivated or wild-harvested ginseng [21,22,23,24,25,26,27]. Plant age modulates the expression of core biosynthetic pathway genes in a tissue-dependent manner, with roots showing increased transcript and metabolite levels over time, whereas stems and leaves exhibit fluctuating or declining trends [21,22,23]. However, nearly all of these findings rely on short-read RNA sequencing or bulk metabolite measurements, which cannot resolve isoform-level dynamics or distinguish alternative splicing (AS) variants that may fine-tune enzyme function [28]. Thus, while the age-dependent transcriptional landscape has been increasingly well characterized, the role of post-transcriptional regulation, particularly AS, in tissue- and germplasm-specific ginsenoside accumulation has received limited attention, prompting the present investigation.

PacBio single-molecule real-time (SMRT) sequencing enables full-length isoform detection and comprehensive AS identification [29,30,31], and AS is increasingly recognized as an important post-transcriptional regulator of plant specialized metabolism. In tea, PacBio Iso-Seq revealed AS as a potential modulator of flavonoid, theanine, and caffeine biosynthesis [31]; in *Panax notoginseng*, combined SMRT and Illumina sequencing uncovered AS events associated with tissue-specific saponin accumulation [32]. For *P. ginseng*, Han et al. (2020) identified AS genes involved in ginsenoside biosynthesis, including HMGR, DXS, and GPS [33], and Zhao et al. (2023) demonstrated that differentially alternatively spliced genes (DAGs) co-localize with quantitative trait loci (QTLs) associated with ginsenoside content [34]. These studies collectively indicate that AS is functionally relevant to ginsenoside biosynthesis, yet direct comparisons between cultivated and wild-simulated ginseng across multiple tissues remain lacking.

In this study, roots, stems, and leaves from 4-year-old cultivated ginseng and 26-year-old wild-simulated ginseng were used as experimental materials. By integrating PacBio full-length transcriptome sequencing and Illumina short-read RNA sequencing, we systematically characterized transcriptomic complexity and ginsenoside-associated co-expression modules. Through quantitative HPLC analysis of ginsenoside content combined with full-length transcript identification, alternative splicing analysis, differential expression profiling, and WGCNA-based co-expression network construction, this study aimed to (i) explore the potential involvement of alternative splicing in enhanced ginsenoside accumulation in wild-simulated ginseng; (ii) identify candidate gene modules and key candidate genes associated with protopanaxadiol-type ginsenoside biosynthesis; and (iii) provide a resource of candidate targets for future functional validation and quality improvement of *Panax ginseng*.

## 2. Materials and Methods

### 2.1. Plant Materials and Sample Collection

Four-year-old cultivated ginseng (CG) and twenty-six-year-old wild-simulated ginseng (WSG) were collected from Fusong County, Jilin Province, China, in July 2025 and authenticated by the Ginseng and Antler Center of Jilin Agricultural University. For each germplasm, three healthy and pest-free plants were selected as biological replicates. Roots, stems, and leaves were separated immediately after harvest, rinsed with sterile water, blotted dry, snap-frozen in liquid nitrogen, and stored at −80 °C until total RNA extraction and ginsenoside analyses. For clarity, tissue samples were designated as follows throughout the manuscript: CL (CG leaf), WL (WSG leaf), CS (CG stem), WS (WSG stem), CR (CG root), and WR (WSG root).

### 2.2. Determination of Total and Monomeric Ginsenoside Contents

#### 2.2.1. Total Ginsenoside Content

Total ginsenoside content was determined according to the Chinese National Standard GB/T 18765-2015 [35] using the vanillin–sulfuric acid colorimetric method with minor modifications. Briefly, approximately 1 g of powdered ginseng sample was subjected to Soxhlet extraction, followed by methanol (analytical grade, CAS 67-56-1; Xilong Scientific Co., Ltd., Shantou, Guangdong, China) extraction and purification with water-saturated n-butanol (analytical grade, CAS 71-36-3; Beijing Chemical Factory, Beijing, China). The final extract was dissolved in methanol and reacted with 8% vanillin-ethanol solution (vanillin, analytical grade, CAS 121-33-5, Sinopharm Chemical Reagent Co., Ltd., Shanghai, China; anhydrous ethanol, analytical grade, CAS 64-17-5; Xilong Scientific Co., Ltd., Shantou, Guangdong, China) and 72% sulfuric acid (analytical grade, CAS 7664-93-9; Xilong Scientific Co., Ltd., Shantou, Guangdong, China). After incubation in a 60 °C water bath for 10 min and cooling on ice, absorbance was measured at 544 nm using a UV–visible spectrophotometer (DU-7500, Beckman Instruments, Fullerton, CA, USA). Ginsenoside Re was used as the reference standard to generate the calibration curve, and total ginsenoside content was calculated accordingly. The vanillin–sulfuric acid assay has been widely used for total saponin quantification in medicinal plants [36].

#### 2.2.2. Monomeric Ginsenoside Content

The contents of eight major ginsenosides (Rb1, Rb2, Rb3, Rc, Rd, Re, Rf, and Rg1) were determined according to GB/T 18765-2015 by high-performance liquid chromatography (HPLC) with minor modifications. Samples were extracted with water-saturated n-butanol after chloroform defatting and dissolved in methanol prior to analysis. Chromatographic separation was performed on a C18 reversed-phase column (4.6 mm × 250 mm, 5 μm) using an acetonitrile–water gradient elution system at a flow rate of 1.0 mL min^−1^. The detection wavelength was set at 203 nm, the column temperature was maintained at 30 °C, and the injection volume was 10–20 μL. Individual ginsenosides were quantified using the external standard method based on peak areas. This method has been widely validated for the simultaneous determination of ginsenosides in *Panax ginseng* [37].

### 2.3. Total RNA Extraction and Quality Assessment

Total RNA was extracted from roots, stems, and leaves using TRIzol^®^ reagent (Invitrogen, Carlsbad, CA, USA) according to the manufacturer’s instructions. RNA concentration and purity were measured using a NanoDrop 2000c spectrophotometer (Thermo Fisher Scientific, Wilmington, DE, USA), and RNA integrity was assessed using an Agilent 2100 Bioanalyzer (Agilent Technologies, Santa Clara, CA, USA). Only RNA samples with an RNA integrity number (RIN) ≥ 7.0 were used for subsequent transcriptome sequencing and quantitative analyses. The TRIzol-based extraction method has been widely applied for obtaining high-quality RNA from *Panax ginseng* tissues suitable for transcriptomic studies [38,39].

### 2.4. PacBio Full-Length Transcriptome Sequencing and Data Analysis

#### 2.4.1. PacBio Library Construction and Sequencing

Equal amounts of total RNA extracted from roots, stems, and leaves of CG.4Y and WSG.26Y were pooled for full-length transcriptome sequencing. Library construction and sequencing were performed by Novogene (Beijing, China). Briefly, poly(A)+ mRNA was enriched using Oligo(dT) magnetic beads and reverse-transcribed into full-length cDNA. Following PCR amplification and SMRTbell adapter ligation, sequencing libraries were constructed and subjected to quality assessment. The libraries were sequenced on the PacBio Sequel II platform (Pacific Biosciences of California, Menlo Park, CA, USA) using single-molecule real-time (SMRT) sequencing technology by Novogene Co., Ltd. (Beijing, China). PacBio Iso-Seq has been widely used for accurate characterization of transcript structures and splice isoforms in plants [40].

#### 2.4.2. PacBio Full-Length Transcriptome Data Processing and Annotation

Raw PacBio sequencing data were processed using the Iso-Seq analysis pipeline implemented in SMRT Link (v25.1) to generate circular consensus sequences (CCSs) and identify full-length non-chimeric (FLNC) reads. High-quality consensus transcripts were obtained after clustering, polishing, and redundancy removal using the Iso-Seq3 workflow and CD-HIT (v4.8.1). Functional annotation was performed against the NR, NT, Swiss-Prot, KEGG, KOG, GO, and Pfam databases using Diamond (v0.8.36), BLAST+ (v2.7.1), and HMMER (v3.1b2). Transcript structures were further characterized by aligning the full-length transcripts to the *P. ginseng* reference genome using pbmm2 (v1.13.1, with minimap2 v2.26), followed by isoform identification using Pigeon (v1.2.0) [41].

### 2.5. Illumina RNA-Seq and Data Analysis

#### 2.5.1. RNA-Seq Library Construction and Sequencing

Total RNA samples from roots, stems, and leaves of CG and WSG were used for RNA sequencing. Three biological replicates were prepared for each tissue and germplasm combination. RNA sequencing libraries were constructed by Novogene (Beijing, China) following the manufacturer’s protocol and sequenced on the Illumina NovaSeq 6000 platform to generate paired-end reads.

#### 2.5.2. RNA-Seq Read Mapping and Differential Expression Analysis

Raw sequencing reads were subjected to quality control using FastQC (v0.11.9) and trimmed with Trimmomatic (v0.39) to remove adapter sequences and low-quality bases. Clean reads were then mapped to the telomere-to-telomere (T2T) *Panax ginseng* reference genome recently assembled by Song et al., which was downloaded from the Figshare repository (https://doi.org/10.6084/m9.figshare.25477741.v2, accessed on 21 August 2025) [42]. Read alignment was performed using HISAT2 (v2.2.1), and transcript abundance was quantified as fragments per kilobase of transcript per million mapped reads (FPKM) using StringTie (v2.2.1). Differentially expressed genes (DEGs) between cultivated ginseng and wild-simulated ginseng were identified using DESeq2 (v1.42.1) with the thresholds of |log_2_ fold change| ≥ 1 and false discovery rate (FDR) < 0.05 [43].

### 2.6. Alternative Splicing Analysis

#### 2.6.1. Iso-Seq-Based Alternative Splicing Event Identification

Full-length transcripts were aligned to the *Panax ginseng* reference genome using GMAP (v2017-06-20) with the parameters --min-trimmed-coverage = 0.85 and --min-identity = 0.90 [44]. Transcript structures were subsequently analyzed using SUPPA2 (V2.3) to identify and classify seven major AS event types, including skipped exon (SE), retained intron (RI), mutually exclusive exon (MXE), alternative donor site (A5SS), alternative acceptor site (A3SS), alternative first exon (AFE), and alternative last exon (ALE) [45].

#### 2.6.2. RNA-Seq-Based Differential Alternative Splicing Analysis

Differential alternative splicing (DAS) analysis was performed using rMATS (v4.3.0), a widely used tool for detecting differential splicing events from RNA-seq data. STAR-aligned BAM files and genome annotation (GTF) files were used as input to quantify reads supporting exon inclusion and exclusion events. Statistical significance was assessed using a likelihood-ratio test, and *p*-values were adjusted using the Benjamini–Hochberg method to obtain false discovery rates (FDRs). Five types of AS events, including SE, RI, MXE, A5SS, and A3SS, were identified. The percent spliced-in (PSI) value was calculated for each splicing event, and significantly differential splicing events between tissues and germplasms were defined using the thresholds of |ΔPSI| ≥ 0.1 and FDR < 0.05. Integrative analyses were subsequently performed to identify genes exhibiting both differential expression and differential alternative splicing [46].

#### 2.6.3. AS Density Calculation

To compare transcript structural complexity between CG and WSG, AS density was defined as the ratio of total AS events to total isoforms (AS per isoform = total AS events/total isoforms). Total AS events included skipped exon (SE), mutually exclusive exon (MX), retained intron (RI), alternative 5′splice site (A5), alternative 3′splice site (A3), alternative first exon (AF), and alternative last exon (AL) events. For ease of visualization, AS events per 1000 isoforms were also calculated.

To account for differences in sequencing depth, FLNC-normalized AS density was calculated as total AS events/FLNC reads × 1,000,000 (AS events per million FLNC reads (APM)). Statistical significance was assessed using a two-sample proportion test (prop.test() in R (v4.4.1)) for AS per isoform and a Poisson rate comparison (poisson.test() in R) for FLNC-normalized AS density. All statistical tests were two-sided, and significance was evaluated at the 95% confidence level.

### 2.7. Weighted Gene Co-Expression Network Analysis (WGCNA)

Weighted gene co-expression network analysis (WGCNA) was performed using the WGCNA package (v1.72-5) in R to identify gene modules associated with ginsenoside accumulation. Genes with low expression levels were removed prior to analysis, and the top 5000 genes ranked by median absolute deviation (MAD) were selected for network construction. A soft-thresholding power was determined using the pickSoftThreshold function to construct a scale-free network. Gene modules were identified using the blockwiseModules function with a minimum module size of 30 and a merge cut height of 0.25. Module eigengenes were subsequently correlated with ginsenoside contents and tissue-specific traits to identify biologically relevant modules. Candidate genes were selected based on their module membership, gene significance values, and expression patterns [47]. The WGCNA pipeline, including module–trait correlation analysis, soft-thresholding power selection, hierarchical clustering of co-expression modules, and topological overlap matrix (TOM)-based network visualization, is shown in Supplementary Figure S1.

### 2.8. Quantitative Real-Time PCR Validation

#### 2.8.1. qRT-PCR Validation of Candidate Genes

Quantitative real-time PCR (qRT-PCR) was performed to validate the expression patterns of selected candidate genes identified from transcriptomic analyses. First-strand cDNA was synthesized using the TransScript^®^ II All-in-One First-Strand cDNA Synthesis SuperMix for qPCR (One-Step gDNA Removal) (TransGen Biotech, Beijing, China) according to the manufacturer’s instructions. Gene-specific primers were designed using Primer3 (v2.5.0) software and are listed in Supplementary Table S1. qRT-PCR was conducted on a QuantStudio™ 3 Real-Time PCR System (Applied Biosystems, Foster City, CA, USA) using PerfectStart^®^ Universal Green qPCR SuperMix (TransGen Biotech, Beijing, China). The thermal cycling conditions consisted of n initial denaturation at 95 °C for 30 s, followed by 40 cycles of 95 °C for 5 s and 60 °C for 30 s. Relative gene expression levels were calculated using the 2^−ΔΔCt^ method, with *PgCYP* serving as the internal reference gene. Each reaction was performed with three biological replicates and three technical replicates. Quantitative real-time PCR is widely used for validating transcriptomic data in plant studies [48,49].

#### 2.8.2. PCR Validation of Alternative Splicing Events

Candidate alternative splicing events identified from the PacBio full-length transcriptome data were validated by RT-PCR using isoform-specific primers (Supplementary Table S2). PCR amplification products were separated by agarose gel electrophoresis to distinguish transcript isoforms based on fragment size. The experimentally observed banding patterns were compared with the predicted transcript structures obtained from Iso-Seq analysis to confirm the presence of alternative splice variants [50].

### 2.9. Functional Enrichment and Statistical Analyses

Gene Ontology (GO; https://geneontology.org/, accessed on 19 December 2025) and Kyoto Encyclopedia of Genes and Genomes (KEGG; https://www.kegg.jp/, accessed on 19 December 2025) enrichment analyses were performed to investigate the biological functions and metabolic pathways associated with differentially expressed genes and co-expression modules. GO enrichment analysis was conducted using the ClusterProfiler package (v4.10.0) in R [51], with significantly enriched terms defined at an adjusted *p*-value < 0.05 (Benjamini–Hochberg correction). KEGG pathway enrichment analysis was performed using the KEGG database [52] via the clusterProfiler::enrichKEGG function, with the *Panax ginseng* genome as the background gene set. Enrichment results were visualized as bubble plots using the ggplot2 package (v3.4.4), where dot size represents gene count and dot color represents the adjusted *p*-value.

Statistical analyses were performed using R (v4.3.1). Data are presented as the mean ± standard deviation (SD) of three biological replicates. Significant differences between groups were evaluated using one-way analysis of variance (ANOVA) [53] followed by Tukey’s multiple comparison test [54] or Student’s *t*-test [55], as appropriate. Differences were considered statistically significant at *p* < 0.05. Prior to parametric testing, normality was assessed using the Shapiro–Wilk test. Given the small sample size (*n* = 3 per group), Shapiro–Wilk results were interpreted in conjunction with Q-Q plots and coefficient of variation (CV < 15% for most saponins), confirming that the data met the assumptions of parametric tests. Homogeneity of variances was evaluated using Levene’s test. For comparisons meeting these assumptions, two-tailed Student’s *t*-tests were performed. In addition, non-parametric Mann–Whitney U tests were applied as a sensitivity analysis, yielding consistent results (Supplementary Table S3).

## 3. Results

### 3.1. Tissue-Specific and Germplasm-Dependent Ginsenoside Accumulation Profiles

**Figure 1:**
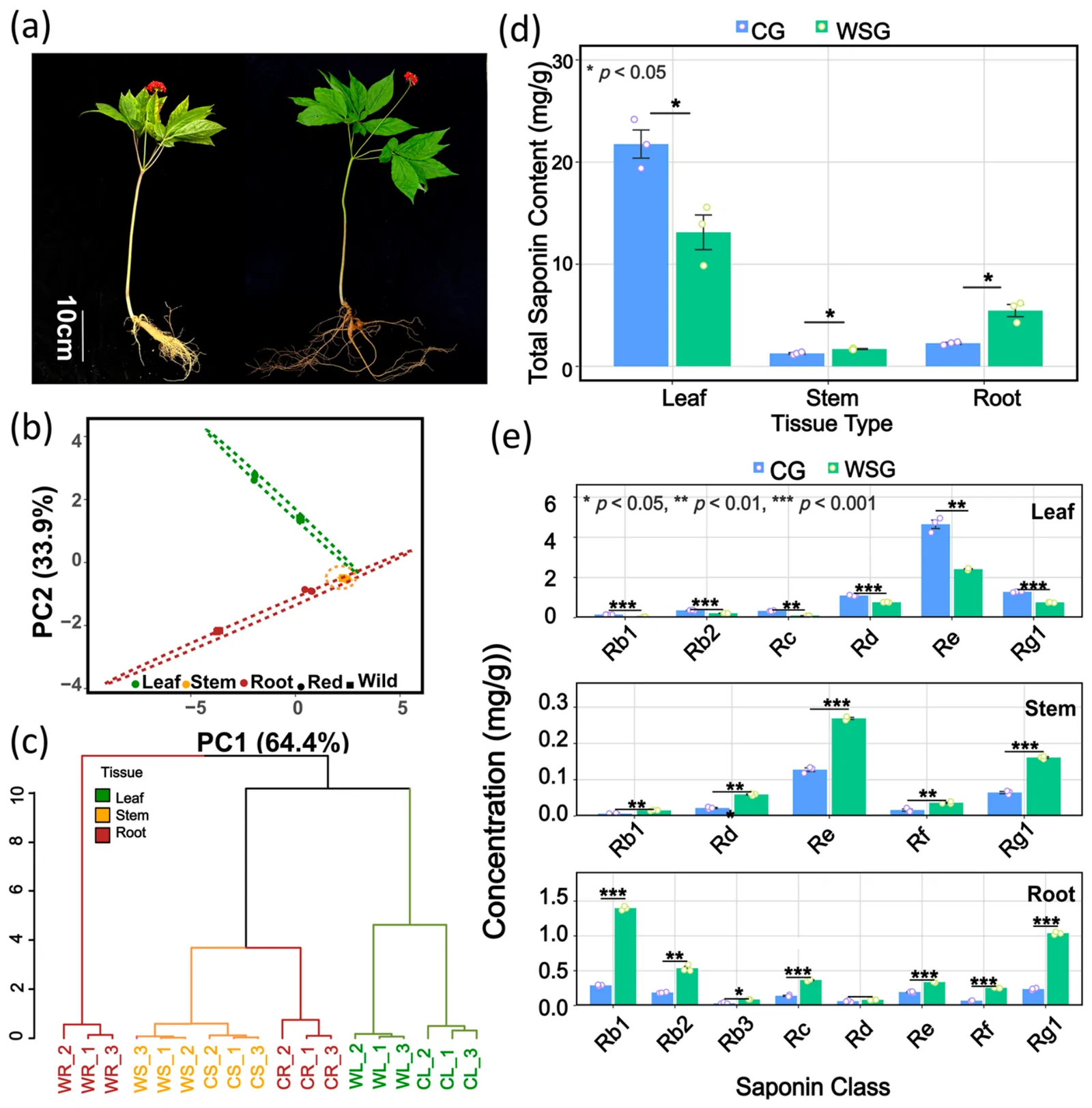
Morphological characteristics and ginsenoside accumulation patterns of 4-year-old cultivated ginseng (CG) and 26-year-old wild-simulated ginseng (WSG): (**a**) Morphology of ginseng samples. Left: CG; Right: WSG. (**b**) Principal component analysis (PCA) based on the contents of eight monomeric ginsenosides. Green, yellow and red represent leaf, stem and root tissues, respectively; circles represent CG and squares represent WSG. (**c**) Hierarchical clustering based on ginsenoside contents. Green, yellow and red branches correspond to leaf, stem and root tissues, respectively; CL (CG leaf), WL (WSG leaf), CS (CG stem), WS (WSG stem), CR (CG root), and WR (WSG root). (**d**) Comparison of total ginsenoside contents among leaf, stem and root tissues. Blue bars: CG; green bars: WSG. (**e**) Monomeric ginsenoside contents in different tissues. The upper, middle and lower panels represent leaf, stem and root tissues, respectively; blue bars: CG; green bars: WSG. Data are presented as the mean ± SD (*n* = 3). Asterisks indicate significant differences between the two germplasms within the same tissue (* *p* < 0.05, ** *p* < 0.01, *** *p* < 0.001).

WSG exhibited multiple branched main roots, abundant fibrous roots, and a dark-yellow epidermis with distinct annular scars, whereas CG had shorter main roots, fewer fibrous roots, and a lighter epidermis (Figure 1a). Principal component analysis (PCA) and hierarchical clustering showed that samples were primarily separated by tissue type, with leaves, stems, and roots forming distinct clusters (Figure 1b,c). Within each tissue, WSG and CG clustered closely, whereas WR were clearly separated from cultivated CR, indicating greater metabolic divergence between the two germplasms in root tissues. This pattern was consistent with the marked difference in total saponin accumulation between WR and CR.

Total saponin content varied markedly among tissues and germplasms (Figure 1d). Leaves contained the highest total saponin levels, followed by roots and stems. In roots, WSG accumulated 5.46 ± 0.98 mg g^−1^ total saponins, which was 2.43-fold higher than that of CG (*p* < 0.05), whereas CG leaves contained significantly more total saponins than WSG leaves (*p* < 0.05).

To further characterize ginsenoside composition, the contents of eight major ginsenosides were quantified by HPLC, and representative chromatograms are provided in Supplementary Figure S2. Monomeric ginsenoside analysis revealed pronounced tissue- and germplasm-specific accumulation patterns (Figure 1e). In leaves, the PPT-type ginsenosides Re and Rg1 predominated and accumulated at significantly higher levels in CG than in WSG (*p* < 0.05). Stems contained much lower ginsenoside levels than leaves and roots, with PPT-type ginsenosides remaining the dominant components. In contrast, roots showed the greatest differences between germplasms. All eight quantified ginsenosides were significantly more abundant in WR than in CR (*p* < 0.05). Among them, Rb1 reached 1.40 ± 0.03 mg/g in WR, representing a 4.83-fold increase over CR, while Rf, Rb2, Rc, and Rg1 were also significantly enriched. These results demonstrate distinct tissue-specific patterns of ginsenoside accumulation and provide the basis for subsequent transcriptomic analyses. Complete statistical details, including means, SDs, 95% confidence intervals, effect sizes (Cohen’s d), and significance levels for all comparisons, are provided in Supplementary Table S3.

### 3.2. Full-Length Transcriptome Sequencing Reveals Distinct Transcript Structural Features in Wild-Simulated Ginseng

**Figure 2:**
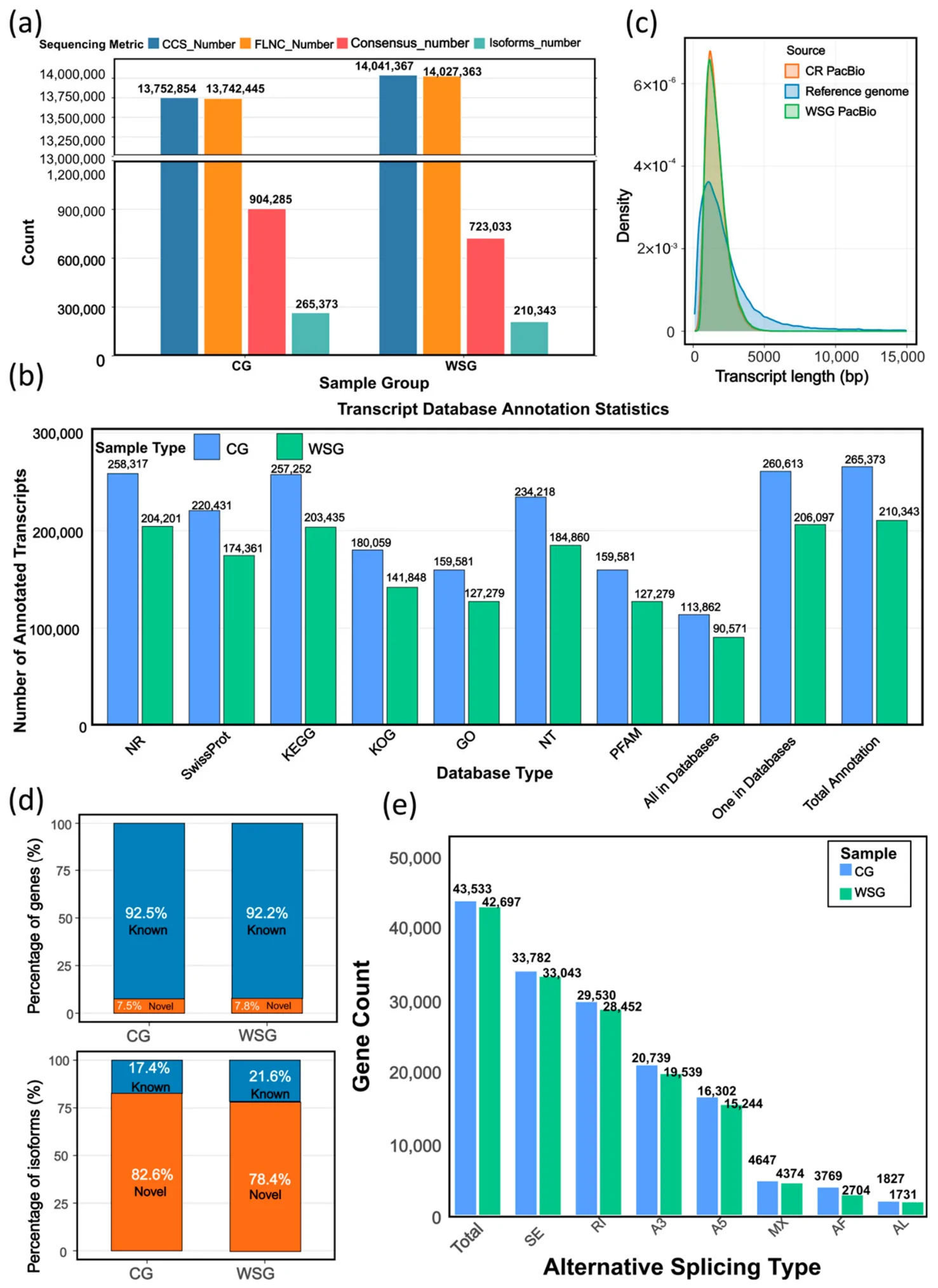
Full-length transcriptome construction and complexity analysis of 4-year-old cultivated ginseng (CG) and 26-year-old wild-simulated ginseng (WSG): (**a**) PacBio sequencing statistics (CCS, FLNC, consensus transcripts, and isoforms). (**b**) Functional annotation against seven databases. (**c**) Density distribution of transcript lengths from CG, WSG, and reference genome. (**d**) Novel genes and novel transcript isoforms identified in CG and WSG. (**e**) Alternative splicing event types (SE, RI, A3, A5, MX, AF, AL) identified by PacBio Iso-Seq.

To characterize transcriptomic complexity, mixed root, stem, and leaf samples from CG and WSG were sequenced using the PacBio Sequel II platform. A total of 27,794,221 circular consensus sequences (CCSs) were generated, of which 99.91% were full-length non-chimeric (FLNC) reads, indicating high sequencing quality (Figure 2a). After clustering, error correction, and redundancy removal, 265,373 and 210,343 non-redundant full-length transcript isoforms were obtained for CG and WSG, respectively (Figure 2a).

Functional annotation against seven public databases (NR, Swiss-Prot, KEGG, KOG, GO, NT, and Pfam) achieved annotation rates of 100% for both transcriptomes, with more than 97% of transcripts annotated in NR and 96% in KEGG (Figure 2b). Most transcripts ranged from 500 to 3000 bp, with the 1000–2000 bp class accounting for approximately 32.5% of all transcripts (Figure 2c). WSG contained a slightly higher proportion of transcripts longer than 3000 bp than CG (5.5% vs. 4.7%). Relative to the reference genome, 3790 novel genes and 164,824 novel transcript isoforms were identified in WSG, compared with 3742 novel genes and 219,207 novel isoforms in CG (Figure 2d).

A total of 12,847 transcription factors belonging to 18 families were predicted, with the C3H, MYB-related, and bHLH families being the most abundant (Supplementary Figure S3). In addition, 519,071 alternative splicing (AS) events involving 45,512 genes were identified, with skipped exon (SE) and retained intron (RI) representing the predominant AS types (32.6% and 32.0%, respectively) (Figure 2e). Although WSG contained fewer AS events than CG (247,203 vs. 271,868), it exhibited a higher AS event density relative to transcript number (Supplementary Table S4).

### 3.3. Multi-Tissue Transcriptome Reveals Tissue and Germplasm-Specific Gene Expression Patterns

**Figure 3:**
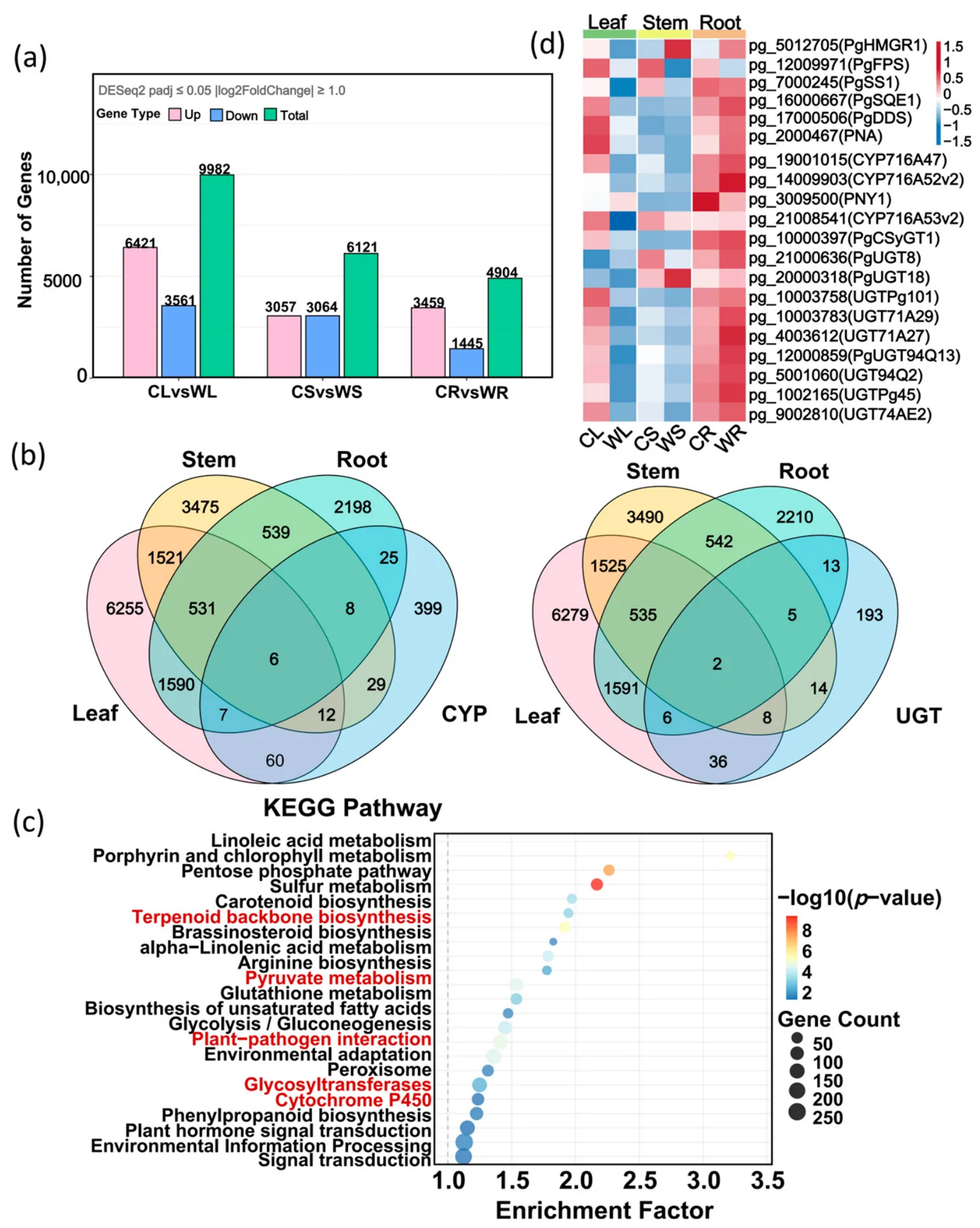
Tissue-specific gene expression and expression patterns of key ginsenoside biosynthetic genes in 4-year-old cultivated ginseng (CG) and 26-year-old wild-simulated ginseng (WSG): (**a**) Statistics of differentially expressed genes (DEGs) between CG and WSG in leaf (CL vs. WL), stem (CS vs. WS), and root (CR vs. WR). DEGs were identified with |log_2_FC| ≥ 1 and FDR < 0.05. Pink, blue and green bars represent upregulated, downregulated and total DEGs, respectively. (**b**) A Venn diagram showing the overlap between differentially expressed genes in leaves (CL vs. WL), stems (CS vs. WS), and roots (CR vs. WR) with genes belonging to the CYP and UGT gene families. (**c**) KEGG pathway enrichment bubble plot of leaf DEGs (CL vs. WL). Dot size indicates gene count; dot color represents −log_10_(*p*-value); the enrichment factor (x-axis) reflects the degree of enrichment, pathways highlighted in red are related to ginsenoside biosynthesis. (**d**) A heatmap of 20 key ginsenoside biosynthetic genes (listed in Supplementary Table S5) across leaf, stem, and root tissues of CG and WSG. Expression values were log_2_(TPM + 1)-transformed and row-standardized by Z-score. Red indicates high expression; blue indicates low expression.

To investigate transcriptional differences between CG and WSG, differentially expressed genes (DEGs) were identified using the criteria of |log_2_FC| ≥ 1 and FDR < 0.05. Leaves (CL vs. WL) contained the largest number of DEGs (9982), followed by stems (CS vs. WS; 6121) and roots (CR vs. WR; 4904) (Figure 3a), indicating that germplasm-dependent transcriptional differences were most pronounced in leaves, while roots exhibited the most conserved expression patterns between the two lines.

The distribution of DEGs within the OSC, CYP, and UGT gene families was further examined (Supplementary Figure S4a, Figure 3b). The OSC family showed relatively few DEGs in leaves (5), stems (3), and roots (3) (Supplementary Figure S4a). In contrast, the CYP family contained the largest number of DEGs, with 85 in leaves, 55 in stems, and 46 in roots. Similarly, the UGT family exhibited more DEGs in leaves (52) than in stems (29) and roots (26), revealing a progressive decline in DEG number from leaves to roots (Figure 3b).

KEGG enrichment analysis showed that leaf DEGs were significantly enriched in terpenoid backbone biosynthesis, cytochrome P450, and glycosyltransferase-related pathways (Figure 3c), whereas root DEGs were mainly enriched in plant hormone signal transduction, MAPK signaling, and the cytochrome P450 pathway (Supplementary Figure S4b).

The expression patterns of key ginsenoside biosynthetic genes (Supplementary Table S5 [16,56,57,58,59,60,61,62,63,64,65,66,67,68,69,70]) are shown in Figure 3d. Most genes exhibited the highest expression in roots, followed by leaves and stems, consistent with the overall distribution of ginsenosides. In roots, *PgDDS*, *PNA*, and *CYP716A47* were more highly expressed in WSG than in CG, whereas the upstream genes *PgSS1* and *PgSQE1* showed relatively stable expression between the two germplasms. In leaves, *HMGR*, *FPS*, *PgDDS*, and *CYP716A47* were more highly expressed in CG than in WSG, while stems displayed generally low expression levels, with relatively higher expression of *CYP716A53v2* and *PgUGT8* in CG.

### 3.4. Tissue-Specific Differential Alternative Splicing Analysis of Key Ginsenoside Biosynthetic Genes

**Figure 4:**
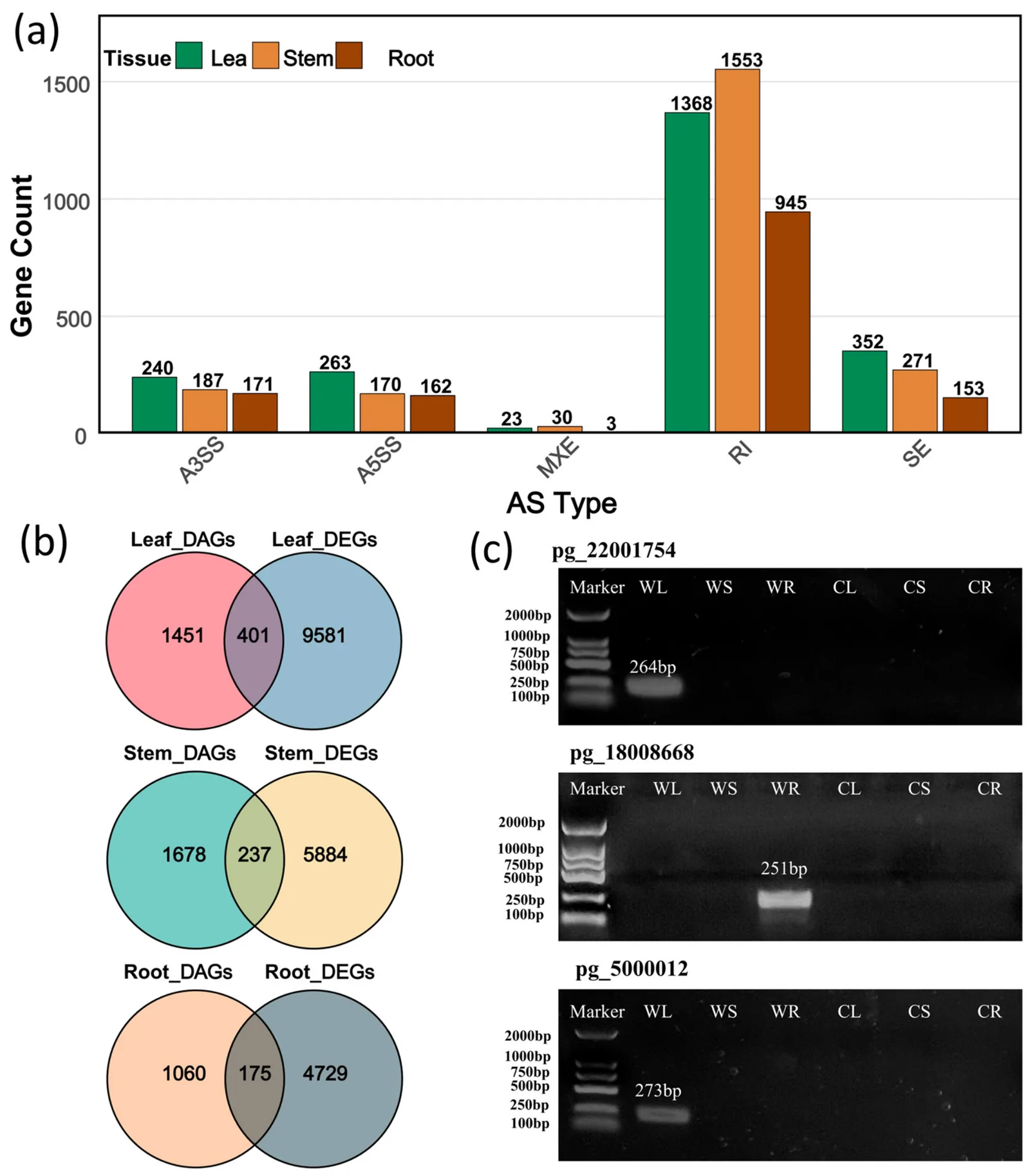
Identification and differential analysis of alternative splicing events in key ginsenoside biosynthetic genes of 4-year-old cultivated ginseng (CG) and 26-year-old wild-simulated ginseng (WSG): (**a**) A bar plot of differential alternative splicing (DAS) event types in leaf, stem, and root tissues. The x-axis shows event types (SE: skipped exon; RI: retained intron; A3SS: alternative 3′ splice site; A5SS: alternative 5′ splice site; MXE: mutually exclusive exons; AFE: alternative first exon; ALE: alternative last exon); the y-axis shows the number of events. (**b**) A Venn diagrams showing the overlap between differentially expressed genes (DEGs) and differentially alternatively spliced genes (DAGs) in leaf, stem, and root tissues. (**c**) PCR validation of representative alternative splicing events in key ginsenoside biosynthetic genes.

To characterize alternative splicing (AS) patterns in CG and WSG, differential alternative splicing (DAS) analysis was performed using |ΔPSI| ≥ 0.1 and FDR < 0.05. A total of 2246, 2211, and 1434 DAS genes (DAGs) were identified in leaves, stems, and roots, respectively (Figure 4a). Retained intron (RI) was the predominant AS event in all tissues, accounting for 61.6%, 71.7%, and 66.9% of DAS events in roots, stems, and leaves, respectively, followed by skipped exon (SE) and alternative 3′/5′ splice sites (A3SS/A5SS) (Supplementary Table S6, Supplementary Material S1).

The overlap between DEGs and DAGs varied across tissues: 401 genes (4.02% of DEGs, 21.65% of DAGs) in leaves, 237 genes (3.87% of DEGs, 12.38% of DAGs) in stems, and 175 genes (3.57% of DEGs, 14.17% of DAGs) in roots. This suggests a partial but tissue-specific coordination between transcriptional and post-transcriptional regulation (Figure 4b). PacBio full-length transcriptome sequencing further revealed distinct isoform structures in several key ginsenoside biosynthetic genes (Supplementary Table S7). Compared with CG, *PgDDS* and *PNA* exhibited fewer AS events in WSG, whereas *PgFPS* displayed increased intron retention together with three novel transcript isoforms. In addition, seven CYP450 and UGT genes were identified as both differentially expressed and differentially spliced. The expression patterns of representative candidate genes were further validated by PCR (Figure 4c; Supplementary Table S8), with the original gel images shown in Supplementary Figure S5.

### 3.5. WGCNA Identifies Candidate Genes Associated with PPD-Type Ginsenoside Accumulation

**Figure 5:**
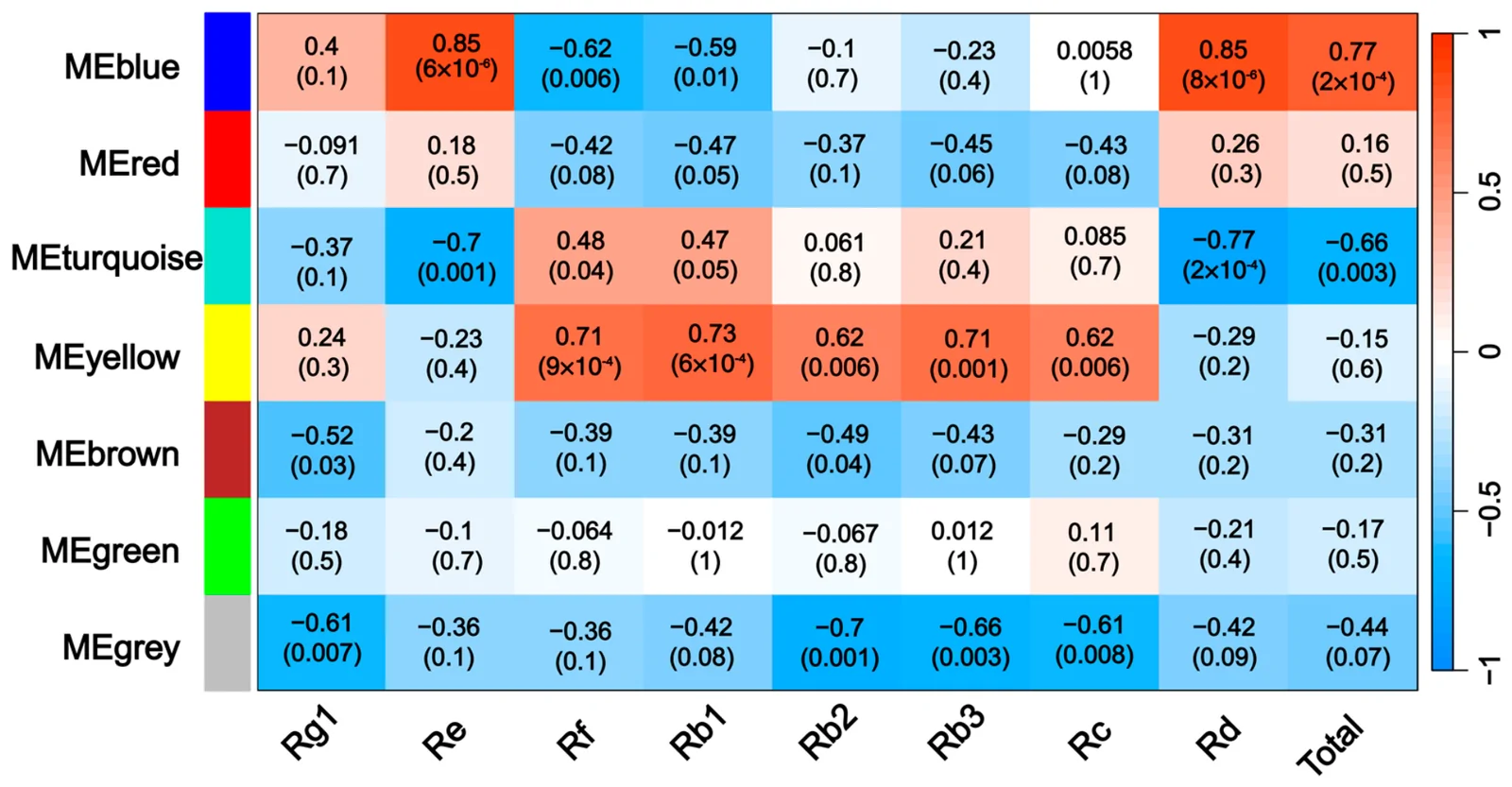
Module–trait correlation analysis between WGCNA-identified co-expression modules and ginsenoside accumulation traits in 4-year-old cultivated ginseng (CG) and 26-year-old wild-simulated ginseng (WSG). Each row represents a co-expression module (ME), and each column represents a ginsenoside trait (eight monomeric ginsenosides and total saponin). The color scale indicates the correlation coefficient (red: positive; blue: negative), with *p*-values shown in parentheses.

To identify co-expression modules associated with ginsenoside accumulation, WGCNA was performed using transcriptome data (FPKM > 1) from 18 samples. The detailed analysis pipeline, including soft-thresholding power selection, hierarchical clustering, and network visualization, is provided in Supplementary Figure S1. Seven co-expression modules were subsequently identified (Figure 5). Correlation analysis between module eigengenes and ginsenoside contents showed that the blue module (845 genes, (Supplementary Material S2)) was strongly positively correlated with Re, Rd, and total saponins (*r* > 0.77, *p* < 0.001). The yellow module (240 genes, (Supplementary Material S2)) showed significant positive correlations with PPD-type ginsenosides (Rb1, Rf, Rb3, and Rc; *r* > 0.62, *p* < 0.01), whereas the turquoise (1642 genes) and gray (1229 genes) modules were negatively correlated with most ginsenosides.

**Figure 6:**
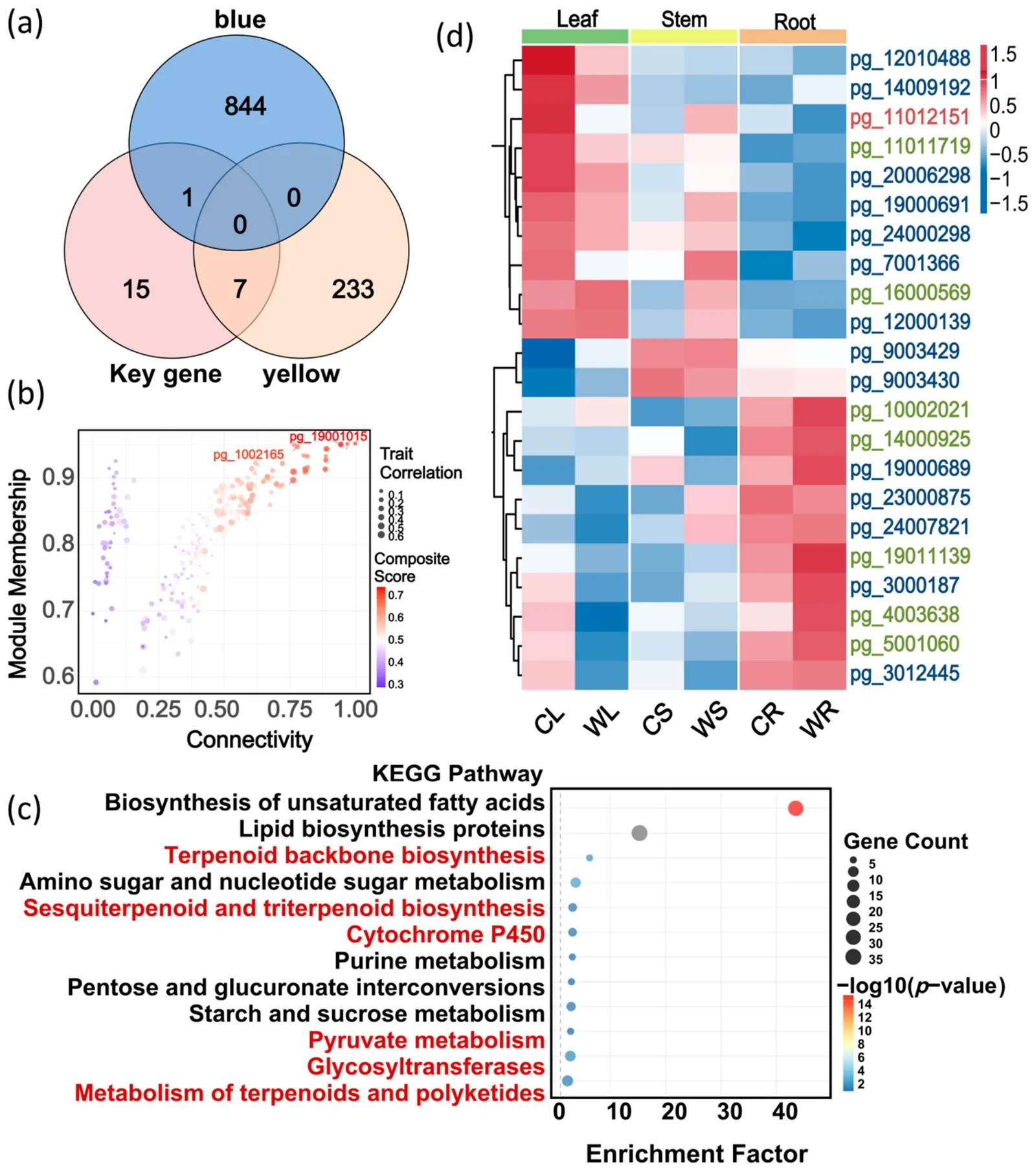
Characterization of the yellow and blue modules and their association with ginsenoside biosynthesis-related genes in 4-year-old cultivated ginseng (CG) and 26-year-old wild-simulated ginseng (WSG): (**a**) A Venn diagram showing the overlap between the blue and yellow modules and 23 experimentally validated ginsenoside biosynthesis genes. (**b**) A dot plot of the top 50 genes in the yellow module ranked by module membership (MM). (**c**) KEGG pathway enrichment bubble plot for genes in the yellow module. Dot size indicates gene count; dot color represents −log_10_(*p*-value); the enrichment factor (x-axis) reflects the degree of enrichment, pathways highlighted in red are related to ginsenoside biosynthesis. (**d**) Composition of predicted ginsenoside biosynthesis-related genes (OSC in red, CYP450 in blue, UGT in green) identified in the yellow and blue modules.

Known ginsenoside biosynthesis genes (Figure 3d) were predominantly assigned to the yellow module, with seven genes mapped to this module compared with only one in the blue module (Figure 6a). The top 50 genes ranked by module membership in the yellow module showed predominant root expression, with pg_19001015 (*CYP716A47*) and pg_1002165 ranking first and third, respectively (Figure 6b). KEGG enrichment analysis indicated that the yellow module was significantly enriched in terpenoid backbone biosynthesis, sesquiterpenoid and triterpenoid biosynthesis, cytochrome P450, glycosyltransferase, and starch/sucrose metabolism pathways (Figure 6c). This module contained one OSC (*PNA*), six CYPs including the functionally validated *CYP716A47*, and nine UGTs, whereas the blue module comprised one OSC (pg_11012151, a putative cycloartenol synthase), nine CYPs, and five UGTs. Among the yellow module genes, pg_14000925 (a UGT) was identified as both differentially expressed and alternatively spliced (Figure 6d; Supplementary Table S8), highlighting its potential as a multi-level regulatory candidate. Differentially expressed genes in these two modules were visualized by heatmap analysis (Figure 6d).

### 3.6. qRT-PCR Analysis Validates the Expression Patterns of Key Ginsenoside Biosynthetic Genes

**Figure 7:**
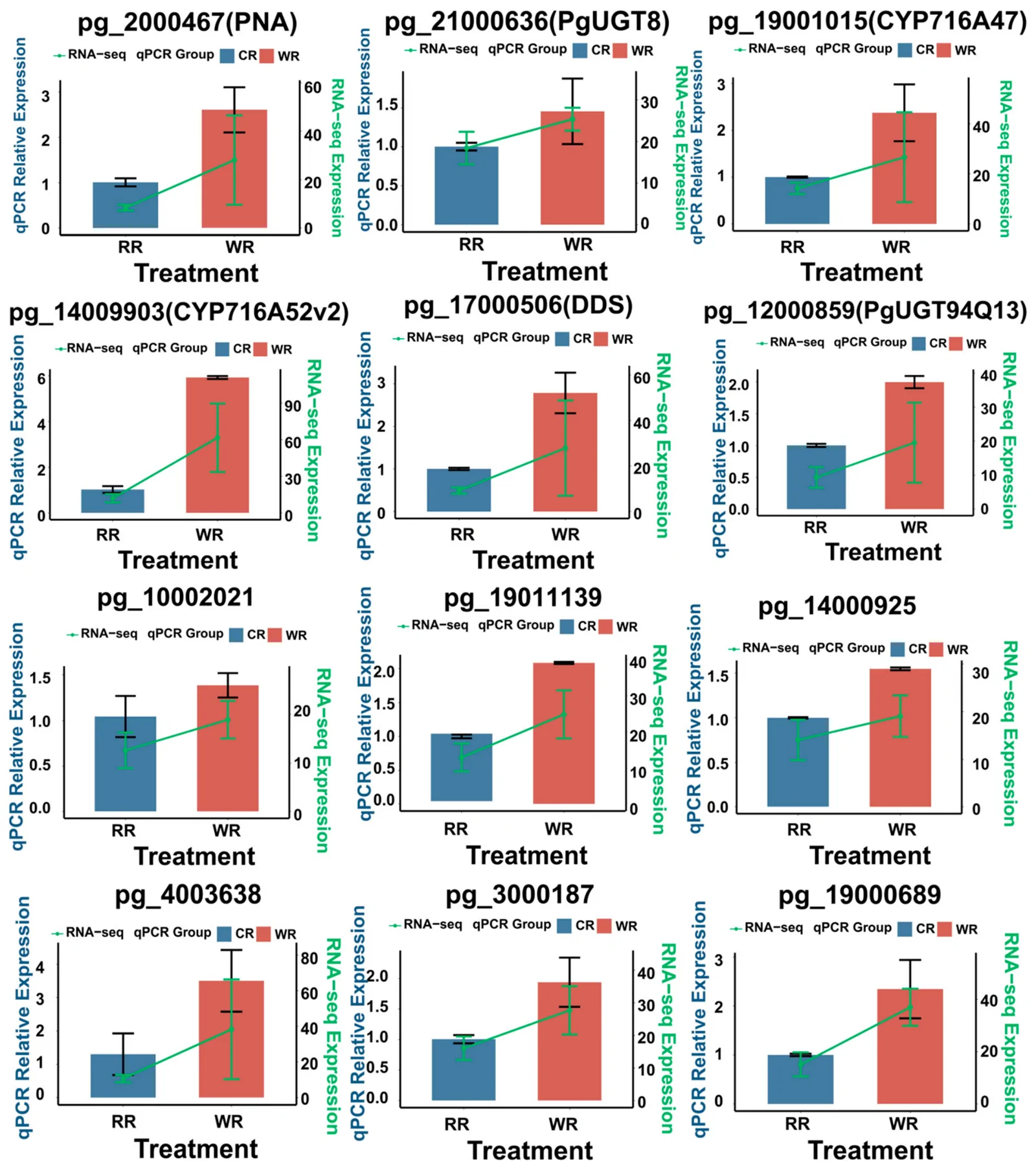
Validation of RNA-seq data by qRT-PCR in root tissues of 4-year-old cultivated ginseng (CG) and 26-year-old wild-simulated ginseng (WSG). Relative expression levels of 12 selected genes (including *CYP716A47*, *UGT94Q13*, and other candidate genes from the yellow module) in roots of CG and WSG: Bar plots (left Y-axis) represent relative expression measured by qRT-PCR, and line plots (right Y-axis) represent FPKM values obtained from RNA-seq. Data are presented as mean ± SD (*n* = 3).

To validate the transcriptome data and confirm the expression patterns of candidate genes, quantitative real-time PCR (qRT-PCR) was performed on selected genes involved in ginsenoside biosynthesis, including key cytochrome P450s, UDP-glycosyltransferases, and several candidate genes identified from the WGCNA yellow module. The qRT-PCR results showed strong concordance with the RNA-Seq data across all tested genes, with comparable expression trends between the two methods. In root tissues, the elevated expression levels of downstream oxidative modification enzymes, particularly *CYP716A47* and *CYP716A53v2*, as well as *UGT94Q13* and *PgUGT45*, were consistently observed in WR relative to CR, corroborating the transcriptomic findings (Figure 7). The qRT-PCR validation for leaf and stem tissues is presented in Supplementary Figures S6 and S7, respectively, and similarly demonstrated good agreement with the RNA-Seq data. Overall, these validation results provide experimental support for the reliability of the transcriptomic analysis and reinforce the proposed regulatory model in which coordinated upregulation of downstream biosynthetic genes contributes to enhanced ginsenoside accumulation in wild-simulated ginseng.

## 4. Discussion

Long-read sequencing has greatly improved plant transcriptomics by enabling accurate reconstruction of full-length transcripts and splice isoforms [71,72]. Recent studies have shown that transcript structural variation contributes to metabolic diversity in medicinal plants, including *Panax* species and other triterpenoid-producing plants [73,74,75]. In this study, WSG exhibited fewer total transcripts but a higher density of alternative splicing (AS) events per transcript than CG, suggesting more diversified transcript regulation under wild-simulated conditions. Numerous novel isoforms identified in WSG belonged to the OSC, CYP450, and UGT gene families, which play central roles in ginsenoside biosynthesis and structural diversification [20,60,76,77]. These findings suggest that transcript structural variation may represent an additional regulatory layer associated with ginsenoside biosynthesis, although its functional significance requires further validation.

Ginsenoside accumulation is influenced by genotype, tissue type, developmental stage, and growth environment [78,79,80,81]. WR accumulated higher levels of total saponins and major PPD-type ginsenosides than CG. However, the increased metabolite accumulation was not accompanied by coordinated upregulation of all biosynthetic genes. Differential expression was mainly observed in downstream modification enzymes, particularly *CYP716A47* and several UGT genes, whereas genes involved in precursor biosynthesis remained relatively stable. *CYP716A47* has been functionally characterized as a key oxidase involved in dammarane-type ginsenoside biosynthesis, while UGTs contribute substantially to ginsenoside structural diversification [16,70]. These results suggest that enhanced ginsenoside accumulation in WSG may depend more on selective regulation of downstream biosynthetic steps than on global pathway activation. Because the cultivated and wild-simulated materials differed simultaneously in plant age, cultivation environment, and potentially genetic background, the relative contributions of these factors could not be fully resolved in the present study. Thus, the observed differences should be interpreted as associations with the two production backgrounds rather than as effects attributable solely to the cultivation system.

Alternative splicing is widespread in plants, but its role in regulating ginsenoside biosynthesis remains poorly understood [82,83,84,85]. Retained intron was the predominant AS event detected in both germplasms, consistent with previous reports [85]. Several key biosynthetic genes displayed distinct splicing patterns between CG and WSG. Compared with CG, WSG showed fewer retained intron events in *PgDDS* and *PNA*, whereas *PgFPS* generated novel transcript isoforms. In addition, several CYP450 and UGT genes were identified as both differentially expressed and differentially spliced. Previous studies have demonstrated that AS contributes to plant specialized metabolism by generating functionally distinct transcript isoforms that fine-tune metabolic pathways [86]. Our results suggest that transcriptional and post-transcriptional regulation may coordinately contribute to ginsenoside biosynthesis. However, the current evidence remains correlational, and further studies are required to clarify the potential roles of specific splice variants in metabolite accumulation.

Gene co-expression network analysis has proven valuable for identifying candidate regulators of specialized metabolism in plants [87]. Previous studies have highlighted the complexity of the ginsenoside biosynthetic network in *Panax ginseng* [42,88]. In the present study, WGCNA identified the yellow module as the gene network most strongly associated with PPD-type ginsenoside accumulation and enriched in CYP450 and UGT family genes. These observations further support the potential importance of downstream modification enzymes in determining ginsenoside composition and provide promising candidates for future functional studies. However, WGCNA is a correlation-based method that identifies co-expression patterns and does not establish regulatory or causal relationships. The candidate genes identified in this module should therefore be prioritized for experimental validation.

## 5. Conclusions

In this study, we integrated PacBio full-length and Illumina short-read transcriptome sequencing to systematically characterize transcriptomic complexity and ginsenoside-associated features in WSG. WSG exhibited a higher AS event-to-isoform ratio than CG, with retained intron and skipped exon representing the predominant AS types. Total saponin content was highest in leaves, followed by roots, and lowest in stems. HPLC analysis revealed that PPT-type ginsenosides (Re and Rg1) predominated in leaves, with significantly higher levels in CG than in WSG. In roots, PPD-type ginsenosides were the dominant forms, and all eight monomeric ginsenosides were significantly more abundant in WSG than in CG. Notably, Rb1 content in WR was 4.83-fold higher than in CR. Transcriptome analysis revealed preferential upregulation of downstream oxidative modification enzymes and glycosyltransferases in WR, whereas upstream biosynthetic genes showed no significant differences. This expression pattern suggests that enhanced ginsenoside accumulation in WSG may be associated with selective regulation of downstream biosynthetic steps. Full-length transcriptome analysis further revealed differential AS patterns in several key biosynthetic genes, and seven CYP/UGT genes were identified as both differentially expressed and differentially spliced, suggesting potential coordination between transcriptional and post-transcriptional regulation. WGCNA identified the yellow module (240 genes) as the module most strongly associated with PPD-type ginsenosides and prioritized six candidate genes for future investigation. Overall, this study provides a valuable resource for understanding transcriptomic regulation associated with ginsenoside accumulation in WSG and establishes a foundation for future hypothesis-driven functional studies. Given the confounding effects of plant age, growth environment, and germplasm background, the present results should be regarded as associative rather than causal. Future studies using age-matched materials, common-garden conditions, and multiple genetic backgrounds are needed to validate these findings.

## Data Availability

The raw sequencing data generated in this study have been deposited in the NCBI Sequence Read Archive (SRA). The Illumina RNA-seq data are available under BioProject accession number PRJNA1483501, and the PacBio full-length transcriptome data are available under BioProject accession number PRJNA1483268.

## Supplementary Materials

The following supporting information can be downloaded at: https://www.mdpi.com/article/10.3390/biology15161367/s1, Supplementary Figures: Figure S1. WGCNA pipeline for constructing gene co-expression networks and identifying trait-associated modules in 4-year-old cultivated ginseng (CG) and 26-year-old wild-simulated ginseng (WSG); Figure S2. Representative HPLC chromatograms of monomeric ginsenosides in leaf, stem, and root tissues; Figure S3. Bar plot of transcription factor families; Figure S4. Tissue-specific expression patterns of differentially expressed genes and their functional enrichment analysis; Figure S5. Original agarose gel electrophoresis images of PCR products used to validate alternative splicing events; Figure S6. Validation of RNA-seq data by qRT-PCR in leaf tissues; Figure S7. Validation of RNA-seq data by qRT-PCR in stem tissues. Supplementary Tables: Table S1. Primers used for qRT-PCR validation of candidate ginsenoside biosynthesis-related genes based on second-generation transcriptome data; Table S2. Primers used for PCR validation of ginsenoside biosynthesis-related genes in alternative splicing analysis; Table S3. Ginsenoside contents in cultivated vs. wild-simulated ginseng tissues; Table S4. Summary of alternative splicing density statistics for CG and WSG; Table S5. Key genes involved in ginsenoside biosynthesis in *Panax ginseng*; Table S6. Summary of differential alternative splicing (DAS) events identified in leaf, stem, and root tissues between CG and WSG; Table S7. Full-length transcriptome-derived alternative splicing events for key ginsenoside biosynthetic genes; Table S8. Differentially expressed and differentially spliced CYP450/UGT genes identified in leaf, stem and root tissues by WGCNA based on second-generation transcriptome data. Supplementary Material S1. Lists of DAS genes and AS events identified in leaf, stem, and root tissues: CL_vs_WL_DAS_events.xlsx, CS_vs_WS_DAS_events.xlsx, CR_vs_WR_DAS_events.xlsx. Supplementary Material S2. Gene lists of yellow and blue modules identified by WGCNA.

## Abbreviations

The following abbreviations are used in this manuscript:

A3SS	Alternative 3′ splice site
A5SS	Alternative 5′ splice
AF	Alternative first exon
AS	Alternative splicing
CCS	Circular consensus sequence
CG	Cultivated ginseng (4-year-old)
CL/S/R	CG leaf/stem/root
CYP	Cytochrome P450
DAG	Differentially alternatively spliced gene
DAS	Differential alternative splicing
DDS	Dammarenediol-II synthase
DEG	Differentially expressed gene
DMAPP	Dimethylallyl diphosphate
FDR	False discovery rate; FLNC, Full-length non-chimeric
FLNC	Full-length non-chimeric
FPKM	Fragments per kilobase of transcript per million mapped reads
GO	Gene Ontology
MAD	Median absolute deviation
MVA	Mevalonate
MXE	Mutually exclusive exon
NR	Non-redundant protein database
NT	Nucleotide database
OSC	Oxidosqualene cyclase
PCA	Principal component analysis
PPD	Protopanaxadiol
PSI	Percent spliced-in
qRT-PCR	Quantitative real-time polymerase chain reaction
RI	Retained intron
SE	Skipped exon
Swiss-Prot	Swiss-Prot protein database
UGT	UDP-dependent glycosyltransferase
WGCNA	Weighted gene co-expression network analysis
WL/S/R	WSG leaf/stem/root
WSG	Wild-simulated ginseng (26-year-old)
