# Supplementary material for: Full-Length Transcriptome Reveals Alternative Splicing Regulation and Key Genes Underlying Differential Ginsenoside Accumulation Between Wild-Simulated and Cultivated Ginseng
Source: Biology (Basel). 2026 Aug 11;15(16):1367. doi: 10.3390/biology15161367 (PMC13509296; doi:10.3390/biology15161367)
Supplement: Supplementary file 1 [file biology-15-01367-s001.zip › 8.11-Supplementary Figures.pdf]

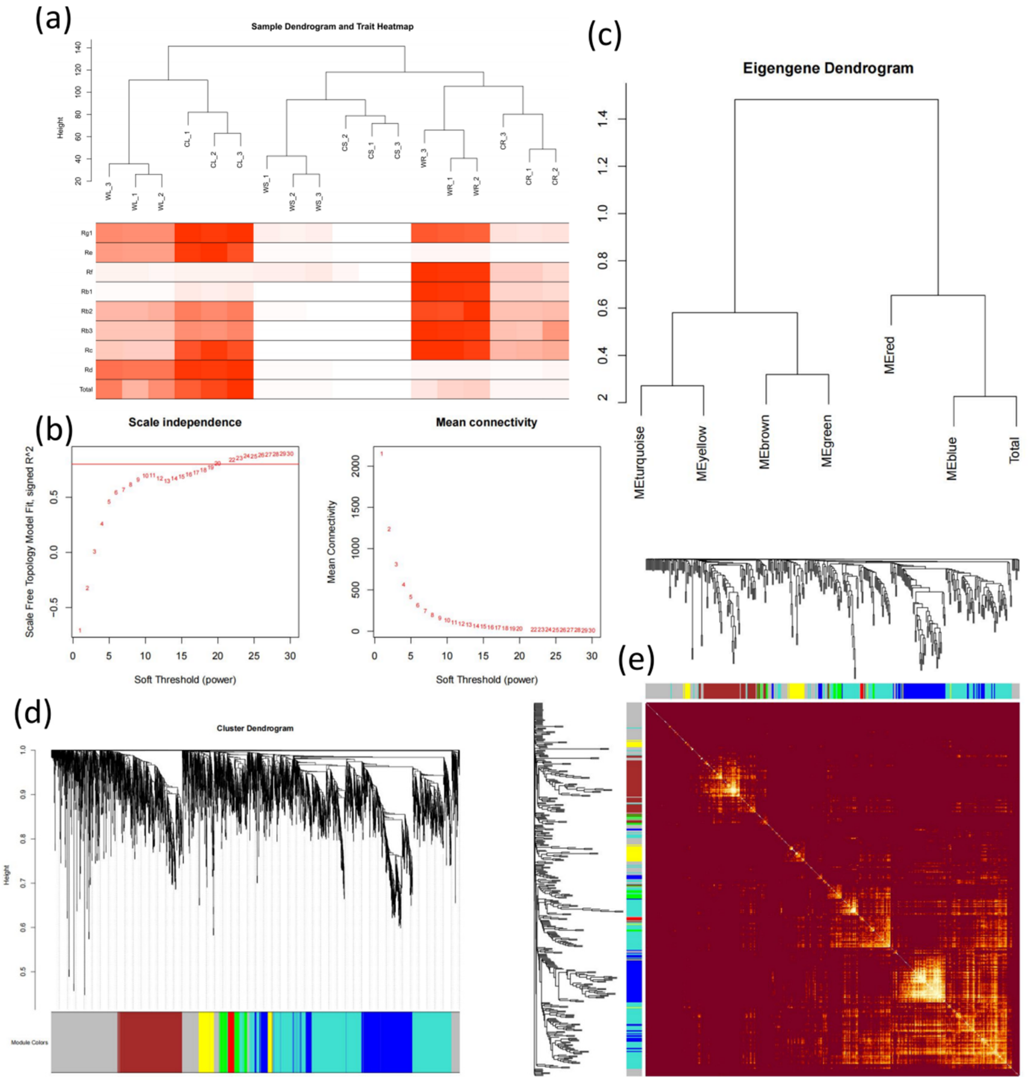

Supplementary Figure S1. WGCNA pipeline for constructing gene co-expression networks and identifying trait-associated modules in 4-year-old cultivated ginseng (CG) and 26-year-old wild-simulated ginseng (WSG): (a) Module–trait correlation heatmap showing associations between co-expression modules and ginsenoside contents; (b) Scale-free topology fit index for soft-thresholding power selection; (c) Hierarchical clustering dendrogram of co-expression modules; (d) Gene dendrogram and module assignment obtained from WGCNA; (e) Weighted gene co-expression network heatmap (topological overlap matrix, TOM) visualizing connectivity patterns among genes.

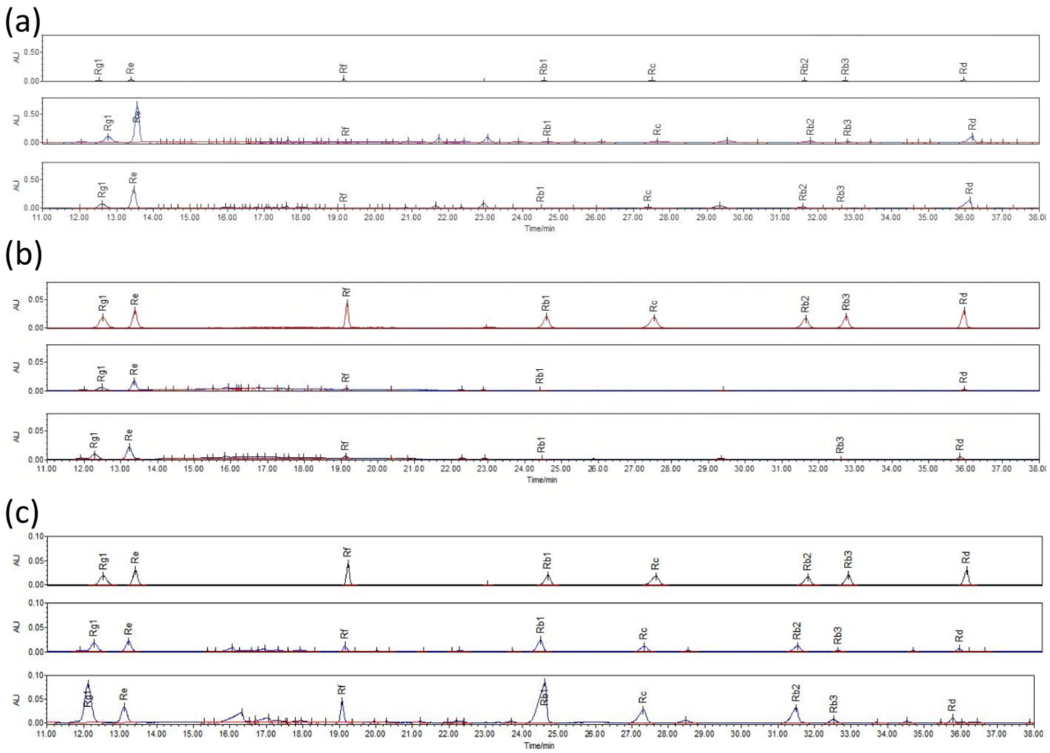

Supplementary Figure S2. Representative HPLC chromatograms of monomeric ginsenosides in leaf (a), stem (b), and root (c) tissues of 4-year-old cultivated ginseng (CG) and 26-year-old wild-simulated ginseng (WSG). From top to bottom: mixed ginsenoside standards, CG, and WSG. Peaks were identified and labeled according to the eight target ginsenosides (Rb1, Rb2, Rb3, Rc, Rd, Re, Rf, and Rg1).

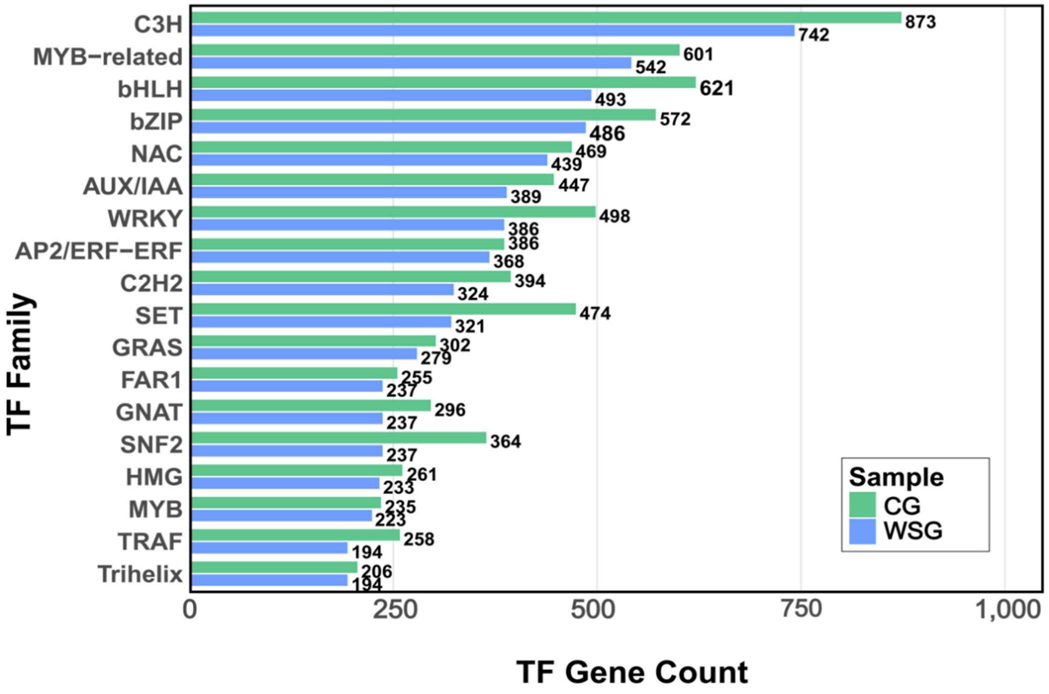

Supplementary Figure S3. Bar plot of transcription factor families identified in CG and WSG transcriptomes. The x-axis shows major transcription factor families; the y-axis shows the number of members in each family. The C3H, MYB-related, and bHLH families were the most abundant.

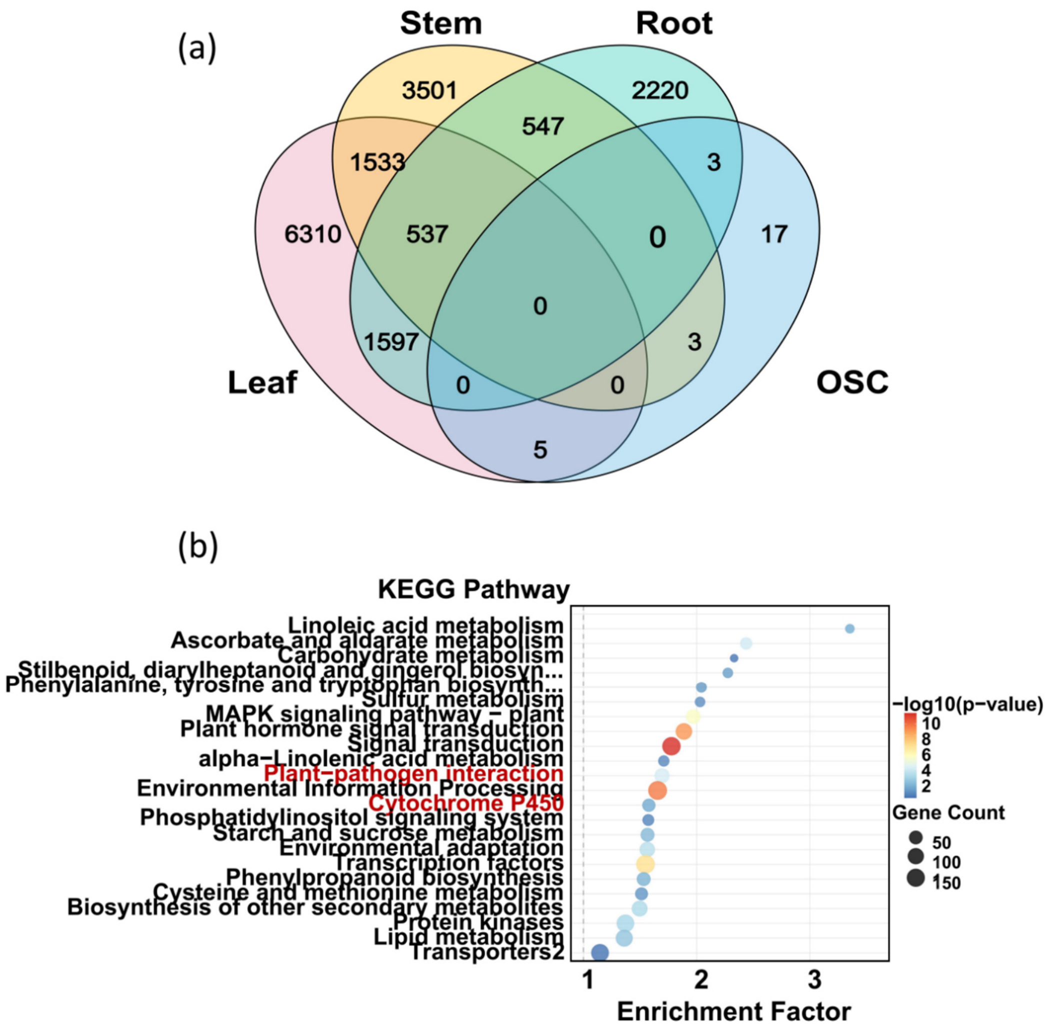

Supplementary Figure S4. Tissue-specific expression patterns of differentially expressed genes and their functional enrichment analysis: (a) Venn diagram showing the overlap between differentially expressed genes in leaves (CL vs WL), stem (CS vs WS),and roots (CR vs WR) with genes belonging to the OSC gene families;(b) KEGG pathway enrichment bubble plot of leaf DEGs (CL vs WL). The x-axis represents the enrichment factor; the y-axis repres ents KEGG pathway names. Dot size indicates the number of genes enriched; dot color represents –log10(*p*-value).

(a)

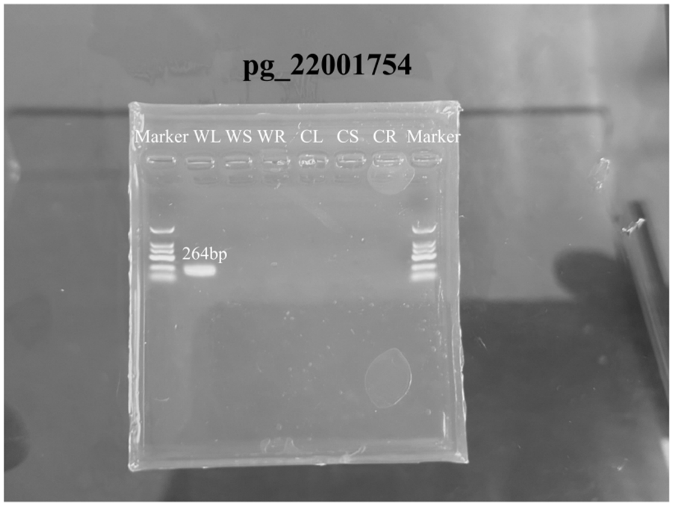
pg_22001754
Marker WL WS WR CL CS CR Marker
264bp

(b)

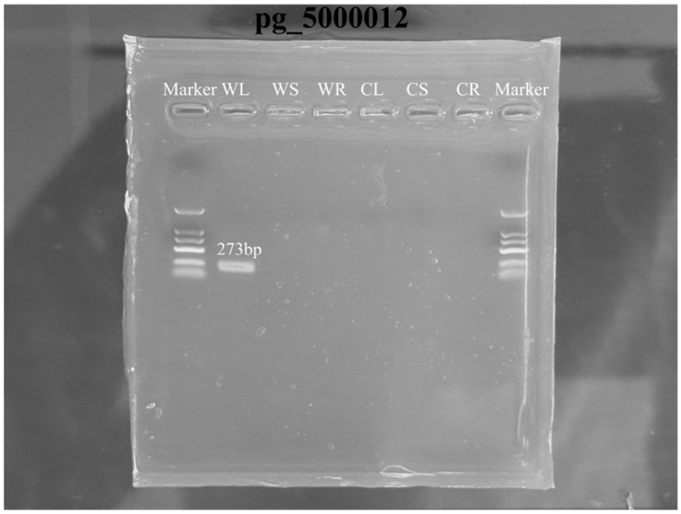
pg_5000012
Marker WL WS WR CL CS CR Marker
273bp

(c)

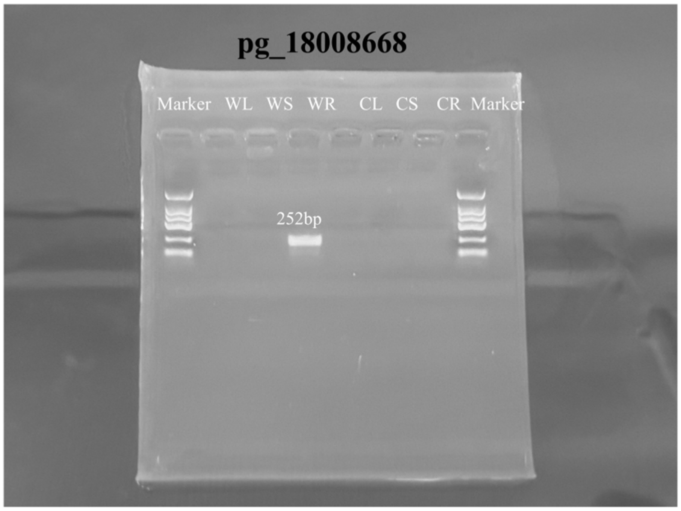
pg_18008668
Marker WL WS WR CL CS CR Marker
252bp

Supplementary Figure S5. Original agarose gel electrophoresis images of PCR products used to validate alternative splicing events. (a) pg_22001754; (b) pg_5000012; (c) pg_18008668.

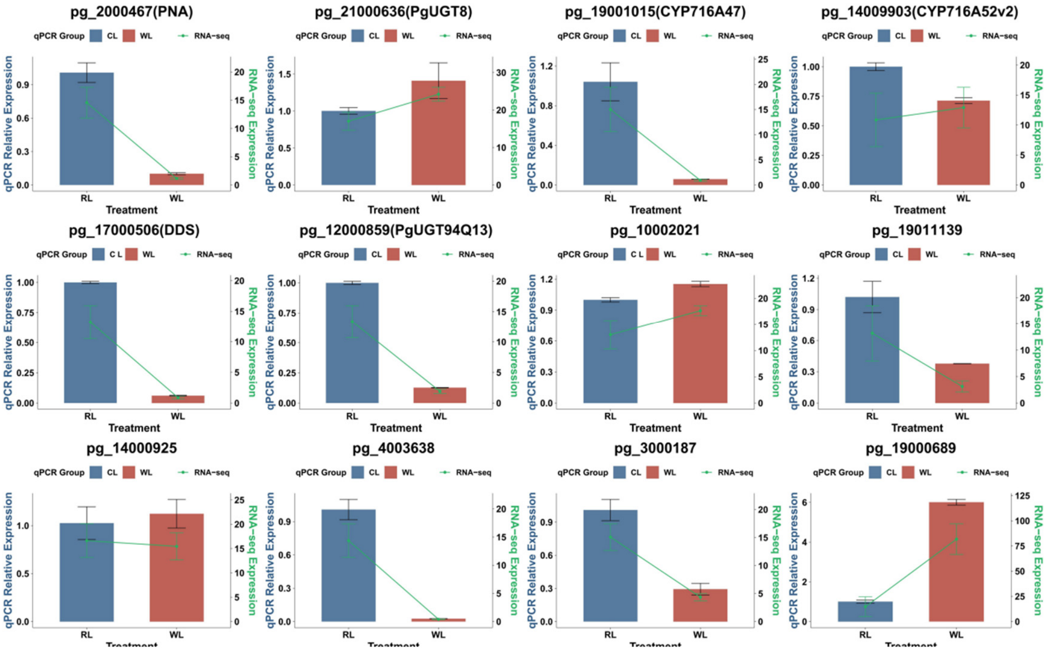

Supplementary Figure S6. Validation of RNA-seq data by qRT-PCR in leaf tissues. Relative expression levels of 12 selected genes in leaf of CG and WSG. Bar plots (left Y-axis) represent relative expression measured by qRT-PCR, and line plots (right Y-axis) represent FPKM values obtained from RNA-seq. Data are presented as mean ± SD (*n* = 3).

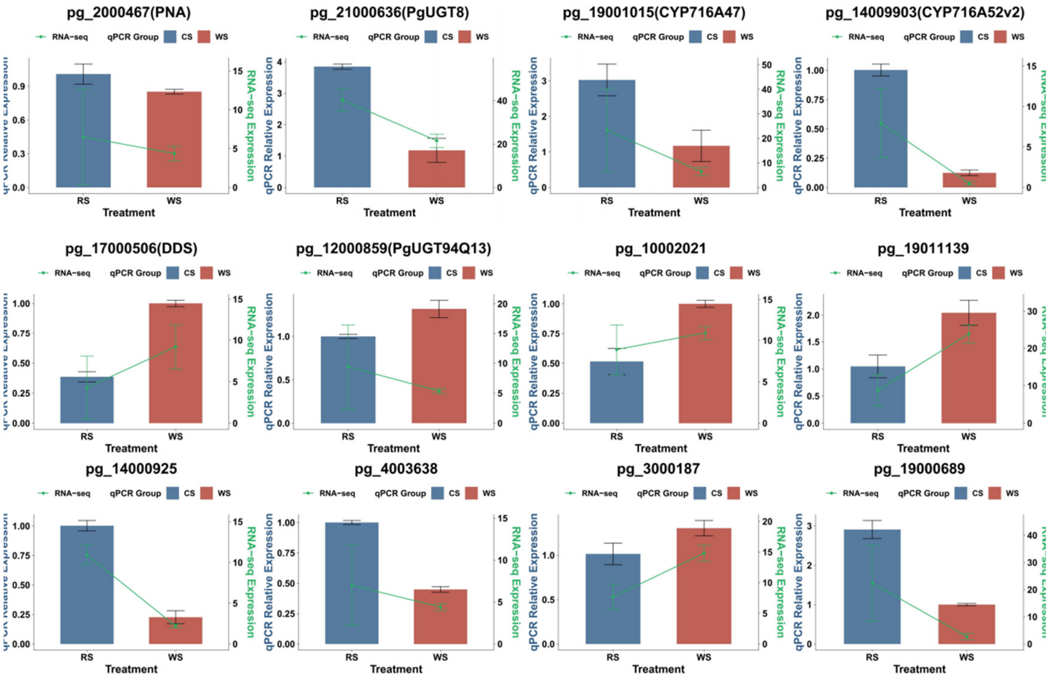

Supplementary Figure S7. Validation of RNA-seq data by qRT-PCR in stem tissues. Relative expression levels of 12 selected genes in stem of CG and WSG. Bar plots (left Y-axis) represent relative expression

measured by qRT-PCR, and line plots (right Y-axis) represent FPKM values obtained from RNA-seq. Data are presented as mean ± SD (n = 3).
